# Effects of Bariatric Surgery on Cardiovascular Disease: A Concise Update of Recent Advances

**DOI:** 10.3389/fcvm.2019.00094

**Published:** 2019-07-10

**Authors:** Toshiki Kuno, Eriko Tanimoto, Sae Morita, Yuichi J. Shimada

**Affiliations:** ^1^Department of Medicine, Mount Sinai Beth Israel Medical Center, New York, NY, United States; ^2^Tokyo Medical and Dental University, Tokyo, Japan; ^3^Division of Cardiology, Department of Medicine, Columbia University Medical Center, New York, NY, United States

**Keywords:** obesity, bariatric surgery, cardiovascular disease, heart failure, coronary artery disease, hypertension, atrial fibrillation, venous thromboembolism

## Abstract

Patients with obesity often have multiple cardiovascular comorbidities as obesity is an established risk factor for various cardiovascular diseases (CVDs)—e. g., heart failure (HF), coronary artery disease (CAD), hypertension, dysrhythmia, and venous thromboembolism. In the United States, obesity is the nationwide public health issue of the day with the prevalence exceeding 30%. It has become a substantial health and financial burden to the society and national healthcare system; the direct cost accounted for 150 billion US dollars in 2014. Lifestyle interventions have been shown to be successful in the short term, however their long-term results are still equivocal likely due to modest weight reduction and high recurrence rates. For instance, the mean weight reduction in a randomized controlled trial of patients with type 2 diabetes mellitus (DM) and either overweight or obesity was 6.0% in the intensive lifestyle modification arm and 3.5% in the control arm. On the contrary, bariatric surgery is known to be the most effective in achieving substantial and long-term weight loss and can prevent the development of CVD risk factors such as DM, hypertension, and dyslipidemia. Bariatric surgery induces prompt weight loss within a few months which lasts for at least 12–18 months, with mean weight loss of ~35% (~70% loss of excess weight), lowering the risk of all-cause mortality, myocardial infarction, and stroke. Furthermore, recent studies demonstrated that bariatric surgery contributed to the reduction of acute care use for HF, CAD, and hypertension. On the other hand, it was reported that bariatric surgery may worsen the control of certain types of CVD (e.g., dysrhythmia), especially in the early postoperative period. Additionally, the notion that being overweight or obese could contribute to higher survival rate in certain populations (e.g., patients with HF)—also known as “obesity paradox”—has been repetitively documented in the past, while most recent investigations suggested that the observed paradox may be attributable to confounding factors including pre-existing comorbidities. Considering the aforementioned advances in the field, this paper reviews a series of recent studies with regard to the short-term and long-term effects of bariatric surgery on various types of CVDs.

## Introduction

Patients with obesity often have multiple cardiovascular comorbidities. This is because obesity is an established risk factor for various cardiovascular diseases (CVDs) including heart failure (HF), coronary artery disease (CAD), hypertension, dysrhythmia, and venous thromboembolism (1). Obesity imposes a significant threat to national public health as well as a substantial economic burden. In the United States (US), statistics in 2014 showed that more than 30% of the population were considered to have obesity—defined as body mass index (BMI) ≥30—resulting in the estimated economic impact of US$150 billion a year (1, 2). Among many approaches to treat severe obesity, studies have demonstrated that bariatric surgery is the most effective and cost-effective treatment, which not only leads to substantial weight loss but also results in higher remission rates of type 2 diabetes mellitus (DM), hypertension, and hyperlipidemia (3–6). More importantly, previous studies have shown that bariatric surgery reduces the overall mortality and the incidence of myocardial infarction and stroke, even though the existence of “obesity paradox” has still been under discussion (7–9). Other studies also revealed that bariatric surgery significantly lowers emergency department visits and hospitalizations due to HF, CAD, and hypertension (10–13). On the other hand, risks of bariatric surgery include low but non-negligible perioperative mortality and CVD complications (e.g., dysrhythmia, venous thromboembolism [VTE]) (1, 14, 15). The main objective of the current review article is to overview the effects of bariatric surgery on the overall and various types of CVD.

## The Socioeconomic Burden of Obesity and Impact on the Overall Health

Being overweight or obese refers to the condition that a person's weight is higher than what is considered as a healthy weight for a given height. BMI has been conventionally used as an indicator to define overweight and obesity. This is a person's weight in kilograms divided by the square of height in meters. For adults, Centers for Disease Control and Prevention and World Health Organization (WHO) define BMI of 18.5 to 25 as the normal range, 25 to 30 as overweight, and 30 or higher as obesity, while age also needs to be taken into consideration for children (16, 17). Although BMI is not a perfect index to assess the relationship between the body weight and health of an individual for its incapability of diagnosing the body fatness, it is still considered the most useful screening tool at an individual level worldwide.

Overweight and obesity are growing global issues, in terms of prevalence, health risks, and socioeconomic impact. Globally, more than 1.9 billion adults aged 18 years and older, nearly 40% of the world's population, were overweight in 2016 (17). Over 650 million people—approximately 13% of the adult population—were obese (17). The worldwide prevalence of obesity nearly tripled between 1975 and 2016 (17). If this rate is kept constant, almost half of the world's adult population will be overweight or obese by 2030 (18). In the US, the prevalence of obesity exceeded 30% in 2015–2016, and 7.7% of the population were severely obese (i.e., BMI >40 kg/m^2^). It is estimated that 42% of the US population will be obese by 2030 at the latest (19).

As another important part of the obesity epidemiology, the prevalence of obesity in a country does not directly correlate with its economic status. When the countries were categorized according to the income level as low-income, lower middle-income, higher middle-income, and high-income, the prevalence of obesity increased up to upper middle-income countries, however the high-income countries came to the second among the 4 categories (20). Low socioeconomic status of individuals has been associated with a higher prevalence of obesity regardless of the nation's economic status, whether it is developing, transitional, or developed (21). Larger disparities in individual access to better quality diet (e.g., fresh fruits, vegetables, and fish) were observed especially in countries with developing or transitional economies as they faced the globalization of food markets (22). This is likely due to greater economic inequality, under-established healthcare systems, and poorer education on diet (22). Development of strategies to address overweight- and obesity-related health problems is therefore warranted worldwide regardless of the nation's economic status.

The associations between excess BMI and substantially shorter healthy and chronic disease-free life expectancy have been well-documented. A meta-analysis on 4 different cohorts from England, Finland, France, and Sweden performed detailed inspection of the WHO's BMI classification on adults from ages 50 to 75 years. This study demonstrated that people with class II obesity could expect to live 6-7 fewer years in good health and 7-9 fewer years without chronic diseases compared to those with normal weight (23).

Overweight and obesity is a complex disease itself and is linked to more deaths worldwide than underweight (20). Obesity is now recognized as the first leading cause of premature mortality followed by cancer and DM (24), and the biggest issue behind this incidence is the association between obesity and CVD. Obesity has long been considered as an established risk factor for CVD. For instance, in a Framingham cohort study, relative weight (i.e., percentage of desirable weight) was found to be positively and independently associated with a 26-year incidence of CAD, stroke, HF, and CVD-related death (25). Recent studies have further investigated how both the duration and degree of obesity affect the risk of different CVDs. Data from the Coronary Artery Risk Development in Young Adults study showed that, for every 2 years lived with obesity, the risk of CVD mortality significantly increased by 7% (26). As for the risk of different types of CVD, another meta-analysis revealed that the relative risk (RR) of stroke with obesity was 1.3, and that of VTE with obesity was 2.4. The risk of HF increased 5% for men and 7% for women per 1 unit increase in BMI (27–30).

Besides physical health problems, the economic impact of obesity has been an important public health issue (2, 14, 31). Obesity imposes large socioeconomic costs not only to the healthcare system but also to the society. Recent studies on the association between BMI and costs attributable to obesity have described that the burden comes in the form of the individual's lost productivity as a result of lost work days, lower productivity at work, mortality, and permanent disability. All of these could lead to a loss of economic growth nationwide (32). Above all, medical costs for obesity-related diseases have been the biggest global concern. Medical costs are typically divided into direct costs and indirect costs. Direct costs include costs for the treatment and management of the diseases—e.g., inpatient and outpatient care. An example of direct non-medical costs is transportation to healthcare providers. Indirect costs include early mortality costs and morbidity costs due to sickness absence and informal care costs. Costs of obesity have been calculated in several studies. In the US, the direct per-capita costs over a lifetime amounted to US$171,482 in 2010, and the total 10-year per-capita costs were predicted to be US $70,200 in 2013 (33). Costs of obesity on the individual, families, and nations have been more enormous than ever, calling not only for global healthcare policy reforms but also for better treatment and preventative interventions on individual basis.

## Effectiveness of Non-invasive Interventions for Obesity

The US National Institutes of Health recommends weight loss for people with BMI >25 kg/m^2^ and either a high waist circumference or >2 risk factors for CVD and other comorbidities (34). Although treatment strategies for obesity vary across regions and there is no authorized body setting the global standard, the first treatment step typically includes diet, lifestyle, exercise, and behavioral modification, and is the basis for every subsequent step. The guidelines recommend these basic treatments plus medication as the next step if weight loss of 5–10% is not achieved within 6 months (35). The last step combines these strategies with bariatric surgery. Overall, non-invasive strategies are the basis of all treatment for obesity.

There have been numerous randomized controlled trials (RCTs) on diet modification revealing effectiveness on weight reduction. An RCT, conducted on an overweight population without traits of metabolic syndrome in China, compared weight reduction with 3 different diets—i.e., low-fat-high-carbohydrate (LF-HC) diet (fat 20%, carbohydrate 66% of total energy intake), middle-fat middle-carbohydrate (MF-MC) diet (fat 30%, carbohydrate 56%), and high-fat low-carbohydrate (HF-LC) diet (fat 40%, carbohydrate 46%). In this study, reduction in body weight was significantly greater in the LF-HC group. After 6 months, weight loss was 0.5 kg greater than that in the MF-MC group and 0.7 kg greater than that in the HF-LC group (36). Glucose metabolism appeared to be the key to these findings. In another study, LF-HC diet contributed to losing weight in normoglycemic individuals, while a diet with a focus on quality of the carbohydrate content (e.g., lower glycemic index, more fiber, and whole grain) worked more effectively on individuals with pre-diabetes regardless of the amount of the caloric intake (37). As reported in a meta-analysis, a reduction in carbohydrate intake appears vital for patients with overweight or obesity and DM. In these populations, a relatively high amount of fat and protein intake was demonstrated to be beneficial for controlling weight and glycemic status (38). Regarding exercise for weight reduction, another systematic review estimated that the combination of physical activity and diet modifications might be more effective than diet modifications alone, likely due to their synergistic effects in improving blood lipids and blood pressure (39).

Weight loss itself is not an ultimate goal for patients with obesity. What are important when evaluating treatment strategies for obesity are their long-term effects on health problems related to obesity—e.g., risk factors for CVD (e.g., hypertension, dyslipidemia, DM) and osteoporosis, and “hard” clinical outcomes such as CVD and fractures (40). Nevertheless, it remains controversial as to whether the aforementioned non-invasive interventions are effective in long-term weight loss and preventing CVD. The key to achieving effective and persistent weight loss is to maintain changes in eating and physical activity behaviors in the long term. However, individuals with obesity often find it difficult. Although prior studies indicated that programs combining diet and exercise produce meaningful weight loss in individuals with obesity soon after the intervention period (41), a meta-analysis of non-invasive interventions has shown that it is challenging to sustain the initial weight loss over 1 year (42). A potential explanation for this observation is that individuals were likely to return, perhaps partially, to their original lifestyle patterns since the lifestyle reform program lasted for a short period of time. Other challenges that patients face while attempting to achieve weight loss include poor access to education from qualified nutrition professionals on certain set of skills and behaviors to achieve weight loss (43). Moreover, several studies investigated adverse effects of non-invasive weight loss interventions. Evidence from cohort studies has shown that deliberate weight loss might be harmful for people who are overweight or obese with BMI ≤35 kg/m^2^ (44). Similarly, adverse consequences were observed in deliberate weight loss in older people and those with CVD who are less remarkably obese (45, 46).

What one should also take into consideration is the limitation of evidence offered by association studies. In contrast to studies reporting “obesity paradox,” a recent analysis found that the risk of premature mortality was lowest at BMIs of 20–25.0 after minimizing confounding and correcting for reverse causality (47). Even RCTs are not without limitations. Numerous RCTs have demonstrated the efficacy of using specific strategies—e.g., calorie reduction, exercise, commercial weight loss programs, popular diets, and prescription weight loss medications (48). Nevertheless, many studies lack generalizability due to their strict eligibility criteria and reliance on frequent personal contact (49). In this context, meta-analysis of RCTs typically provides the most robust evidence to examine the effects of intentional weight loss in adults with obesity. A comprehensive meta-analysis has reported that diets low in fat were associated with a 18% RR reduction in premature mortality over a median follow-up duration of 2 years, corresponding to 6 fewer deaths per 1,000 participants (49). The same study concluded that liquid diets, non-prescription diet pills, and popular diets were not associated with successful weight loss (49). Moreover, those who successfully lost weight were less likely to report consumption of such foods and products as compared to those who did not lose weight (49).

## Effects of Bariatric Surgery on CVD Risk Factors, Myocardial Infarction, Stroke, and Mortality

The rest of the present review will focus on bariatric surgery as it has been shown to be the most effective strategy for weight reduction and is superior to lifestyle and pharmacological interventions. A number of meta-analyses have revealed that bariatric surgery results in a large short-term weight loss (~41 kg in 30 days) (50, 51). The percentage of complete resolution was 77% in DM and 62% in hypertension, and that of substantial improvement in hyperlipidemia was ≥70% (50, 51). With regard to the long-term effects, a meta-analysis of RCTs with 2 years of follow-up also demonstrated that bariatric surgery leads to substantial weight loss (~26 kg) and DM remission (3). Another study revealed its capacity to improve the control of CVD risk factors with the remission rate of 73% in DM, 63% in hypertension, and 65% in hyperlipidemia (14). Further, in a large prospective cohort study, bariatric surgery was shown to reduce the composite of mortality, myocardial infarction, and stroke (hazard ratio [HR] 0.67) (8). Regarding the long-term effectiveness of bariatric surgery on mortality, a retrospective study (median follow-up of 7.1 years) has shown that bariatric surgery may decrease mortality by 40%, reducing mortality due to CAD by 56%, due to DM by 92%, and due to cancer by 60% (52). A cost-effectiveness analysis of bariatric surgery estimated that it would save US$4,970 direct costs and US$10,960 including indirect costs after 5 years (53).

## Obesity, HF, and Bariatric Surgery

HF is a global pandemic affecting 5.7 million adults and produces 1 million hospitalization annually in the US (54, 55). HF-related health expenditures are already considerable—US$30.7 billion in 2012—and estimated to increase dramatically with an aging population to $69.8 billion in 2030 (56). Notably, obesity is an independent risk factor for multiple chronic CV conditions (e.g., hypertension, CAD, left ventricular hypertrophy), which may eventually lead to the development HF. Indeed, as high as 40% of patients hospitalized for HF exacerbation are obese (57). Patients with obesity tend to have larger left ventricular (LV) mass and wall thickness, both of which result in higher LV diastolic filling pressure (58, 59), while the association between obesity and systolic function remains controversial (57, 60). As a possible mechanism of HF in patients with obesity, the concept recognized as “obesity cardiomyopathy” has recently been introduced, proposing that excessive epicardial fat may exert direct cardiotoxicity (61, 62). Additionally, hyperinsulinemia and activation of neurohormonal system including the renin-angiotensin-aldosterone axis may play a role in the development of HF and exacerbation in patients with obesity (60).

Considering the potentially significant contribution of obesity to HF development, weight reduction achieved by bariatric surgery could reduce the risk of incident HF (i.e., primary prevention). A nationwide cohort study of 47,859 patients with obesity reported that the overall incidence of HF was 5 times lower among patients who underwent bariatric surgery than the non-surgical patients. This finding was consistent in all age groups and in all groups with existing comorbidities such as CAD, hypertension, and DM (63). Another cohort study of 3,448 patients with obesity suggested that, after follow-up of up to 8 years, patients who had bariatric surgery had a reduced risk of incident HF (67 cases among the surgical patients vs. 121 cases among the non-surgical patients) and lower risk of death from HF (64).

With regard to secondary prevention of HF (i.e., prevention of morbidities in patients who have already developed HF), previous studies have suggested that weight reduction may be beneficial to reverse LV remodeling (65). Therefore, at least theoretically, bariatric surgery may be effective in reducing LV wall thickness and mass, LV filling pressures, and New York Heart Association functional class, especially in patients with HF with preserved ejection fraction (HFpEF) for whom no interventions have been proven to have mortality benefit (66, 67). Indeed, a few small studies suggested that LV wall thickness, mass, and diastolic function improve after bariatric surgery. For example, a study of 12 patients with HFpEF and obesity reported that LV wall thickness and mass regressed significantly after bariatric surgery. The relative wall thickness regressed from 0.44 at baseline to 0.42 at 3 months and to 0.39 at 6 months, and patients experienced fewer HF symptoms postoperatively (68). Another study suggested that bariatric surgery can improve LV diastolic functions (DF) and thus lead to better control of diastolic HF. In this study, the investigators assessed LVDF by mitral peak early and atrial velocities, E-deceleration time, and pulmonary vein S, D, and A reversal velocities in 60 female patients with obesity at 6 months after bariatric surgery. They observed that LVDF improved and LV mass as well as wall thickness decreased (69). Thus far, the most robust evidence comes from a recent study that has evaluated the association of substantial weight loss, achieved with bariatric surgery, on the rate HF exacerbation in patients with obesity and HF (12). This was a self-controlled case series study of 1,664 patients with obesity and HF who underwent bariatric surgery, using the population-based databases that captured all emergency department (ED) visits and hospitalizations in the 3 states in the US. Compared to the risk of an ED visit or hospitalization for HF exacerbation during the reference period (14.4%), the rate was significantly lower for 2 years after bariatric surgery (8.7%; adjusted odds ratio (aOR) 0.56). This corresponds to >40% reduction in the risk of HF exacerbation after bariatric surgery. Yet, it remains unknown whether bariatric surgery offers mortality benefit in patients with obesity with HF—if it does, bariatric surgery may become the first and only intervention to improve mortality in patients with HFpEF (70).

## Effects of Bariatric Surgery on Stable Angina Pectoris

Stable angina pectoris (SAP) affects ~8.2 million adults in the US (71), and ~22,000 patients are hospitalized for SAP annually (72). Obesity may worsen the control of SAP through accelerating coronary atherosclerosis, endothelial dysfunction, and LV hypertrophy, and increasing demand for cardiac output (10, 50). With regard to the primary prevention of angina, a nationwide retrospective cohort study revealed, with a mean follow-up of 3.4 years, strong protective associations between bariatric surgery and the incidence of angina (HR 0.59) (73). Another population-based study of patients with obesity and type 2 DM involving 2,580 patients who underwent bariatric surgery and 13,371 patients who did not showed that bariatric surgery was associated with a significant reduction in the incidence of angina (0.4% of the surgical group vs. 1.8% of the non-surgical group) with a median follow-up of 21.2 months (74).

In terms of secondary prevention of SAP, little has been investigated as to whether weight reduction contributes to the improvement of SAP-related morbidity. In a recent self-controlled case series study of 953 obese adults with SAP who underwent bariatric surgery, it was elucidated that the rate of hospitalizations for SAP was reduced by approximately two-thirds after bariatric surgery (10). Possible explanations for the reduction of the rate of the SAP-related morbidity include the improvement of LV hypertrophy, blood pressure, glucose control, and lipid profile through substantial weight loss. Additionally, bariatric surgery, at least theoretically, may have favorable effects on endothelial function, systemic inflammation, and oxidative stress (50). Some of these factors would have contributed to the reduction in the risk of developing new-onset angina and to the better control of angina-related symptoms.

## Bariatric Surgery and Hypertension

Hypertension is also an essential public health challenge worldwide. In the US, approximately one third of total population have hypertension and the estimated direct and indirect costs have reached as much as US$49 billion (75). Annually, 1 million people are sent to emergency department and half a million patients are hospitalized due to hypertension (71). Obesity is associated with elevated risk of uncontrolled hypertension. Inadequate hypertension control in obesity has emerged as a vital issue (76). With regard to primary prevention of hypertension, a meta-analysis with a median follow-up of 36 months showed that the risk of incident hypertension decreased after bariatric surgery (RR 0.54) (77). This study also investigated the relationship between reduction in BMI and the risk over time. There was a rapid reduction in the risk of incident hypertension as BMI decreased during the first 18–20 months. The risk reduction reached a plateau at approximately 20 months after surgery.

With regard to the effects of large weight reduction on the control of hypertension, it has been known to decrease blood pressure and even result in remission of hypertension. For instance, a randomized trial of 96 patients with obesity and hypertension examined if an improvement of hypertension control would occur, defined as a reduction of ≥30% of the total number of antihypertensive medications while maintaining controlled office blood pressure. Such improvement was observed in 84% of patients in the bariatric surgery group compared with 13% in the non-surgical group. Moreover, 51% of patients in the bariatric surgery group showed remission of hypertension, while no patients in the non-surgical group were free of antihypertensive drugs at 12 months (78). A retrospective review of 88 patient with type 2 DM and hypertension, treated with bariatric surgery, reported that a reduction of the number of antihypertensive medications was observed in 50 patients (2.2 medications at baseline to 1.2 medications per patient at 1 month after surgery) (79). Furthermore, a systematic review demonstrated that bariatric surgery resulted in resolution of hypertension in 58% of patients at 1-year and that 75% of patients experienced resolution or improvement of hypertension (80). So far, however, few studies have examined the effect of bariatric surgery on the prevention of hypertension-related acute care use (e.g., ED visits and unplanned hospitalizations) (3, 81, 82). In this context, a recent population-based study gives light to the effectiveness of substantial weight loss on hypertension-related adverse events (11). In this study of 980 patients with obesity and hypertension who underwent bariatric surgery, ~18% of the study population had an acute care use for hypertension during the 1-year pre-surgery period. Within the first year after bariatric surgery, the rate significantly decreased to ~12%. Overall, growing evidence supports that bariatric surgery is highly effective in preventing the development of new-onset hypertension, and is also a promising approach for avoiding hypertension-related acute care use among patients with hypertension.

## Effects of Bariatric Surgery on Dysrhythmia and Venous Thromboembolism

It has been better recognized in the recent years that the relationship between obesity and dysrhythmia—e.g., atrial fibrillation (AF)—is not as simple as it has been thought. Obesity is known to be one of the potent risk factors for developing dysrhythmias. Moreover, it has been suggested in several prospective cohort studies that substantial weight loss may reduce the incidence of new-onset AF (83–85). For example, a study reported that patient in the bariatric surgery group had a 29% lower risk of being diagnosed with first time AF during inpatient hospital admission or hospital-based outpatient consultations (12.4% of the surgery group vs. 16.8% of the non-surgical group) during a median follow-up of 19 years (86). The effect was larger in younger individuals and patients with higher diastolic blood pressure (86). Another study of 5,044 patients observed that the incidence of new-onset AF was significantly lower in the surgical group compared to the non-surgical group (0.8 vs. 2.9%). In the surgical group, higher preoperative BMI and older age were risk factors for the development of new-onset AF (87). Given these prior reports, bariatric surgery has been expected to reduce the risk of AF-related morbidities (e.g., ED visits, hospitalizations) among patients with pre-existing AF. Nonetheless, in a recent study of patients with obesity and AF who underwent bariatric surgery, the risk of AF-related acute care use transiently increased for a few months after the surgery. This would be probably attributable to a number of factors including postoperative inflammation, anemia due to blood loss, electrolyte abnormalities, and infection (13, 88). Further, the risk did come back to the preoperative level but did not go any lower (88), questioning the expected favorable effects of bariatric surgery on the control of dysrhythmias and related morbidities. A plausible explanation would be that adverse remodeling and fibrotic changes of the left atrium may be advanced and irreversible even after a substantial weight reduction with bariatric surgery in some patients with long-standing morbid obesity (13, 15, 88). Another potentially underlying mechanism would be that chronically impaired absorption of nutrients and the resultant anemia and electrolyte deficiencies after bariatric surgery may lead to increased AF episodes and more severe symptoms in the long term (15).

Similar relationship was observed with regard to obesity, VTE, and bariatric surgery. Obesity is an established, and perhaps one of the strongest, risk factor for developing VTE (13, 89). Therefore, it would be natural to hypothesize that a substantial weight loss would reduce the risk of VTE-related acute care use. Yet, a recent study elucidated that the risk of acute care use for VTE was transiently increased after bariatric surgery in the immediate postoperative period, and was back to the baseline level within a few months but not further reduced during a 2-year follow-up (13). This observation would be largely due to immobility during the postoperative period. These findings provide important insights on the differential effects of bariatric surgery on certain types of CVD. Bariatric surgery is an invasive procedure that is typically performed in patients with severe obesity, and does not always offer favorable effects on some specific types of CVD even when obesity is an established risk factor.

## Bariatric Surgery on the Overall CVD and Cost

CVD is the leading public health problem in the US, affecting approximately one third of population in the US. In 2010, 4.4 ED visits and 5.8 million hospitalization were observed (1), and the direct and indirect costs of CVD reached to US$316 billion (90). Thus far, we have discussed the favorable effects of substantial weight loss, achieved by bariatric surgery, on the morbidities related to various types of CVD—e.g., myocardial infarction, stroke, HF, SAP, and hypertension. On the other hand, we have also displayed that bariatric surgery may not be effective in reducing the risk of acute care use due to certain types of CVD (e.g., dysrhythmia, VTE)—it may even worsen the control of these CVDs in the early postoperative period. In this context, a comprehensive examination on the effects of bariatric surgery on the overall CVD has recently been performed (13). In this cohort study that analyzed 11,106 adults with obesity and CVD who underwent bariatric surgery, the risk of CVD-related acute care use (i.e., ED visit, unplanned hospitalization) reduced from 21 to 19% (aOR 0.91; *P* = 0.002) during the first postoperative year and to 17% during the second postoperative year (aOR 0.84; *P* < 0.001). The effect size was smaller in this study on the overall CVD compared to the aforementioned studies with a focus on the individual CVDs showing 40–70% risk reduction. This is probably because the benefit on ischemic CVDs and HF was partially offset by the transient unfavorable effects on dysrhythmia and VTE.

## Obesity Paradox

In the prior sections, we have discussed the effects of substantial weight loss achieved by bariatric surgery on the individual types of CVD. In addition, the effects were generally favorable when the overall CVD were examined. The inferences from these reports may appear contradictory to what is called “obesity paradox”—a well-recognized medical hypothesis that there may be an inverse relationship between obesity and CV prognosis in long-term follow-up (91–95). For example, a previous study reported that individuals with mild obesity (BMI 30–34.9 kg/m^2^) counterintuitively had better prognosis after ST-elevation segment myocardial infarction than patients with BMI <25 kg/m^2^ (92). Similarly, patients with overweight and mild obesity were shown to have lower risk of stroke and HF (93, 94). The mechanism of obesity paradox has yet to be fully understood. It might be due to certain confounding factors or related to intrinsic limitations of BMI (96), as it does not necessarily reflect distribution or amount of body fat (94). Genetic factors may also play a role in obesity paradox. Lean patients with CVD are reported to have genetic disposition that is etiologically different from patients with obesity (97, 98). Another study indicated potential influence of unintentional weight loss and underlying diseases, greater metabolic reserve, less cachexia, and protective cytokines (97).

Recently, however, the pendulum has begun to swing to the other direction—several studies have argued against obesity paradox among patients with CVD (99). Moreover, the series of studies on the CV effects of bariatric surgery has elucidated overall favorable effects as discussed above (10–13, 15, 100). This would not be a surprise as patients who undergo bariatric surgery typically have severe obesity where there is no “J-curve” theory reported in terms of CVD risk in this BMI range. In other words, the “obesity paradox” mainly applies to patients with overweight and mild obesity, which is a distinctively different patient population from those who are candidates for bariatric surgery (1, 92).

## Non-CV Morbidities of Bariatric Surgery

Although bariatric surgery can be a powerful method for treating patients with severe obesity and CVD to prevent CVD-related morbidities and to reduce subsequent healthcare costs, it is an intra-abdominal surgery that does involve the risk of perioperative complications. Non-CV short-term complications of bariatric surgery include low but not negligible mortality and respiratory failure, wound infection, pneumonia, and anemia due to blood loss. In the long term, patients who underwent bariatric surgery may experience weight regain, recurrence of DM, bone loss, as well as vitamin and mineral deficiency and resultant chronic anemia. Moreover, in some studies, there was a signal suggesting worsening control of substance abuse and depression—mortality rate related to accidents and suicide increased by 58% in surgery group (1, 14, 15). Therefore, the decision of performing bariatric surgery should be made carefully while taking into consideration these risks and patient's preferences as well as physical and mental tendencies.

## Conclusions

Thanks to the recent advancements in the understanding of the CV effects of bariatric surgery, it has been elucidated that substantial weight loss achieved with bariatric surgery can prevent morbidities related to a variety of CVDs such as myocardial infarction, stroke, HF, SAP, and hypertension. On the other hand, it has also been exhibited that bariatric surgery may not be effective in certain types of CVDs—e.g., dysryhtmias, VTE. Combined with the existing data on the short-term and long-term non-CV complications of bariatric surgery, the recent series of studies on the CV effects of bariatric surgery adds to the body of our knowledge by providing more precise information on its risk-benefit balance in patients with CVD. This would in turn help physicians and patients make informed and individualized decisions on the selection of treatment options for severe obesity—e.g., lifestyle modifications, pharmacological treatment, and bariatric surgery—especially when there is a co-existing CVD.

## Author Contributions

YS contributed conception and constructing the overall structure and contents. TK and YS wrote the first draft of the manuscript. ET and SM wrote sections of the manuscript. All authors contributed to manuscript revision, read and approved the submitted version.

### Conflict of Interest Statement

The authors declare that the research was conducted in the absence of any commercial or financial relationships that could be construed as a potential conflict of interest.
